# Sexual dimorphism in SLE: above and beyond sex hormones

**DOI:** 10.1177/0961203318815768

**Published:** 2018-12-01

**Authors:** E.A.A. Christou, A. Banos, D. Kosmara, GK Bertsias, DT Boumpas

**Affiliations:** 1 Laboratory of Inflammation and Autoimmunity, Biomedical Research Foundation of the Academy of Athens, Athens, Greece; 2 Department of Rheumatology, Clinical Immunology and Allergy, University of Crete School of Medicine, Heraklion, Greece; 3 Laboratory of Autoimmunity and Inflammation, Institute of Molecular Biology and Biotechnology, Foundation for Research & Technology – Hellas (FORTH), Heraklion, Greece; 4 Joint Rheumatology Program, 4th Department of Internal Medicine, Attikon University Hospital, National and Kapodistrian University of Athens Medical School, Athens, Greece; 5 Rheumatology–Clinical immunology Unit, Medical School, University of Cyprus, Nicosia, Cyprus

**Keywords:** Female predominance, lupus, genetic, epigenetic, microbiota, hormones

## Abstract

Systemic lupus erythematosus (SLE) is characterized by aberrant production of auto-antibodies and a sexual dimorphism both in the phenotypic expression and frequency of the disease between males and females. The striking female predominance was initially attributed primarily to sex hormones. However, recent data challenge this simplistic view and point more towards genetic and epigenetic factors accounting for this difference. More specifically, several SLE-associated single-nucleotide polymorphisms (SNPs) have been found to play an important role in the gender predilection in SLE. Their effect is mediated through their involvement in sex-hormone and immune system signalling and dysregulation of the expression of genes and miRNAs pertinent to the immune system. Additionally, the genetic factors are interchangeably associated with epigenetic modifications such as DNA methylation and histone modification, thus revealing a highly complex network of responsible mechanisms. Of importance, disturbance in the epigenetic process of X chromosome inactivation in females as well as in rare X chromosome abnormalities leads to increased expression of X-linked immune-related genes and miRNAs, which might predispose females to SLE. Microbiota dysbiosis has also been implicated in the sexual dimorphism by the production of oestrogens within the gut and the regulation of oestrogen-responsive immune-related genes. Sexual dimorphism in SLE is an area of active research, and elucidation of its molecular basis may facilitate ongoing efforts towards personalized care.

## Introduction

Systemic lupus erythematosus (SLE) is a chronic autoimmune disease characterized by the production of multiple autoantibodies directed against cellular components, which trigger an immune-mediated injury and damage to multiple organs. SLE is a genetically complex disease in which environmental, genetic and epigenetic factors lead to perturbation of complex biological networks, thus culminating in diverse clinical phenotypes of varying severity.^1,2^

SLE is characterized by a much higher prevalence in women than men, with a female-to-male ratio ranging from 8:1 to 15:1 in adults.^3^ In general, the difference is greater in premenopausal as compared to postmenopausal or prepubertal females. Female-to-male ratio is decreased in both the younger-onset (<18 years) and older-onset (>50 years) groups (4.7:1 and 5:1, respectively).^4^ Peak incidence of SLE also differs, with male patients affected predominantly between 15 and 28 years of age and female patients between 22 and 35 years of age.^4^

The reported female predominance in SLE points to a strong sex hormone effect.^3^ However, recent data demonstrate that sex hormones are not the only cause of the female predisposition in SLE. Herein, we provide a current update on the presumed mechanisms of the sexual dimorphism in the disease beyond sex hormones.

## Proposed mechanisms for the gender bias in SLE

### Genetics

The pathogenesis of the female predisposition in SLE cannot be fully explained by the hormonal differences between genders. The fact that prepubertal girls and postmenopausal women still develop SLE at a higher rate than age-matched males brings genetic factors into play (Table 1).^3^ Among them several SLE-associated single-nucleotide polymorphisms (SNPs) have been identified as candidate contributors to the disease's sexual pattern. These SNPs refer to genes involved in sex hormone and immune system signalling as well as in epigenetic mechanisms (Table 2).

**Table 1:** Genetic mechanisms contributing to SLE sexual dimorphism

Genetic mechanisms/factors	Description
SLE-associated SNPs	• Affect epigenetic mechanisms (DNA methylation, histone modification) • Dysregulate the expression of X-linked genes and SLE risk genes • Influence sex hormone signalling • Impact on immune system function
Aberrant gene and miRNA expression between sexes	Results from: • oestrogen and/or IFN effect • VGLL3 transcription factor upregulation • eQTL effect of SLE-associated SNPs • epigenetic mechanisms
IFN-responsive genes and oestrogen interaction	• Leads to oestrogen and IFN loop
X chromosome complement	• Upregulates the immune-related X-linked genes or miRNAs • Result in skewed X inactivation in Klinefelter syndrome • Contributes to X chromosome inactivation escape

SLE: systemic lupus erythematosus; IFN: interferon; eQTL: expression quantitative trait loci; SNP: single-nucleotide polymorphism.

**Table 2:** SLE-associated genes possibly implicated in gender predilection and their functions

Gene	Chromosome	Function	Gender predilection	Reference
FCER1A	1q23	Plays an important role in asthma and in allergic disease	Female	^12^
Osteopontin	4q22	Involved in bone formation and in immune system function	Male	^6^
HLA Region 1	6p21	Endogenous antigen presentation to the immune system	Male	^5^
HLA Region 2	6p21	Exogenous antigen presentation to the immune system	Male	^5^
IRF5	7q32	Regulates IFN production. Activated by TLR and MyD88 pathways	Male	^5^
TLR7	Xp22	Recognizes viral ssRNA and induces type I IFN production	Possibly male	^7,10^
TLR8	Xp22	Recognizes viral ssRNA and induces type I IFN production	Female	^9,10^
UBE2L3	22q11	Participates in immune system function and in sex hormone signalling	Possibly female	^18,19^
NR3C4	Xq12	Participates in sex hormone signalling	Possibly female	^17^
IRAK1^a^	Xq28	Involved in epigenetic mechanisms	Possibly female	^15^
MECP2^a^	Xq28	Involved in epigenetic mechanisms	Possibly female	^16^
KIAA1542	11p15	Possibly involved in IFN responses	Female	^5,11^
CXorf21	Xp21	Not known	Possibly female	^13^

IFN: interferon; ssRNA: single-stranded RNA.

a In the same linkage-disequilibrium block.

#### SLE-associated SNPs and immune system

Men are suggested to require a higher cumulative genetic load than women in order to develop SLE.^5^ They are more likely to carry SLE high-risk alleles in autosomal genes with significant functions in the immune system such as the HLA, IRF5 and osteopontin (SPP1) genes.^5,6^ The X-linked TLR7 SNP rs3853839 is significantly associated with SLE, with this association being stronger in male than in female patients.^7^ Furthermore, TLR7 G-allele carriers exhibit increased expression of the gene and a subsequently higher interferon-alpha (IFN-α) production compared with C-allele carriers.^7^ A weak association of G-allele of TLR8 SNP rs3764879 with SLE occurrence has been observed in women compared to men (*P* = 0.05, OR 1.36, 95% CI 0.99–1.86).^8^ Mouse studies further support the potential pathogenic role of TLR8 SNP for SLE females indicating that TLR8 escapes X inactivation in 564Igi female mice, thus enhancing its expression and leading to increased IFN-α production.^9^ Both the TLR7 and TLR8 are phylogenetically related and recognize viral single-stranded RNA (ssRNA), inducing an innate immune system response.^10^ The SNP rs4963128 of KIAA1542 is associated with greater SLE development in women than in men and is in high linkage disequilibrium with the rs702966 SNP in IRF7, which is a regulator of IFN production.^5,11^ The rs2298804 of FCER1A, which codes for the high-affinity IgE receptor and plays an important role in asthma and in allergic disease, was more strongly associated with SLE in Chinese women than men.^12^ Moreover, the SNP rs887369, which is located in close proximity to the X-linked gene CXorf21, is associated with the development of SLE.^13^ CXorf21 gene function remains unknown, and there is some evidence supporting an increased expression of CXorf21 in B cells and monocytes in SLE patients.^14^ Further studies are needed to evaluate a possible role for CXorf21 in the immune system. Importantly, CXorf21 escapes X-inactivation, thus being a possible imbalanced gene-dose candidate for the development of SLE in females.^13^

#### SLE-associated SNPs and epigenetics

Several SNPs in the X-linked IRAK1/MECP2 locus have been associated with SLE, by affecting the epigenetic mechanisms of DNA methylation, miRNA expression and histone modification.^15,16^ SNPs in this locus upregulate MECP2 isoform 2 mRNA expression in stimulated T cells.^15^ Also, studies using MECP2 transgenic mouse confirmed the impact of upregulated MECP2 on all three mechanisms of epigenetic regulation highlighting the complex pathogenic function of SLE-associated SNPs in this locus.^15^

#### SLE-associated SNPs and sex hormone signalling

The sexual dimorphism in SLE could be mediated by gene variants that regulate sex hormone signalling. The CD44 (rs2732547) region of the androgen receptor NR3C4 is significantly associated with SLE and reduces the binding activity of the androgen receptor, constituting a potential pathogenetic mechanism for sexual dimorphism in SLE.^17^ Moreover, the discovery of the SLE risk gene UBE2L3, an E2 ubiquitin-conjugating enzyme with a role in immune system function as well as in sex hormone signalling, highlights the complexity of the involved mechanisms.^18,19^

### Gene expression

#### Disturbance of gene expression

In SLE, several genes pertinent to the immune system are differentially expressed between sexes. It is reported that the expression of 26 genes in B cells is downregulated in SLE women compared to SLE men.^20^ Importantly, three of the above-mentioned genes (LTF, CAMP and DEVA4) are implicated in the immune response and are downregulated after oestrogen treatment in mice.^20^ The ZAS3 locus, which encodes a transcriptional molecule that regulates inflammatory responses, is also upregulated in SLE patients following oestrogen treatment.^21^ Recently, the transcription factor VGLL3 was found to be upregulated in female compared to male SLE patients.^22^ VGLL3 regulates the expression of several genes that are pertinent to SLE, such as BAFF, MMP9, IL-7 and ICAM-1, and promotes type I interferon responses via sex hormone-independent mechanisms.^22^ On the other end of the spectrum, there is evidence for an oestrogen and IFN loop, thus highlighting the complexity of regulatory mechanisms. In lupus-prone NZB*NZW F1 female mice, IFN-α or IFN-γ act synergistically with oestrogen to increase the expression of oestrogen (E2)- and IFN-responsive genes.^23^ Both IFN-inducible genes and oestrogens have a pathogenetic role in SLE.^23^ The IRF5 gene, which is associated with increased risk of SLE development, is upregulated in lupus-prone mice both in steady conditions and following oestrogen treatment and regulates type 1 IFN expression.^24^

An expression profiling study identified six genes that were differentially expressed in SLE males versus females but not in healthy males versus females, highlighting a potential role in disease sexual dimorphism.^25^ The particular gene products are involved in multiple biological procedures, such as transcriptional regulation and DNA damage repair (SMC1A), lipoprotein particles catabolism (APOE), glutathione biosynthesis and metabolism (OPLAH), correct composition of bone and cartilage matrix (ARSD), whereas two of these genes are noncoding genes (MTCO2 and FRG1B).^25^

#### eQTL effect of the SLE-associated SNPs

The reported difference in gene expression between sexes might be an effect of the SLE-associated SNPs. For example, six autosomal genes were found to be differentially expressed in healthy females compared to healthy males according to the genotype of SLE/primary Sjogren's syndrome (pSS)-associated SNPs.^26^ These sex-specific effects of SLE/pSS-associated SNPs in gene expression, also known as expression quantitative trait loci (eQTL) effects, provide an explanation for the increased SLE risk in females compared to males.^26^ Regarding the reported eQTL effect on gene expression, it is also unclear whether it is mediated by oestrogens, as the majority of genes that are differentially expressed between sexes are not under the influence of sex hormones.^26^

#### miRNAs and epigenetics

Several miRNAs are differentially expressed in SLE patients compared to healthy individuals.^27^ miRNAs are small RNA molecules that control the gene expression and have a close relation with epigenetic mechanisms.^28^ miRNAs affect the epigenetic mechanisms of DNA methylation and histone modification and at the same time the miRNA expression is under epigenetic control.^28^ For instance, miR148a, which contributes to DNA hypomethylation of SLE CD4 ^+^ T cells, was found to be upregulated in SLE patients compared to healthy individuals, whereas miR125a and miR146a, negative regulators of the inflammatory cytokine RANTES and the IFN-α pathway respectively, were found to be decreased.^3^ Oestrogen treatment in mice alters miR148a, miR125a, and miR146a expression.^3^ Despite the existing data, it remains elusive whether oestrogen action is the true cause of the gender-related differential expression of the allegedly oestrogen-regulated genes in SLE.

### X chromosome gene dosage

#### X chromosome abnormalities and SLE risk

**Figure 1:**
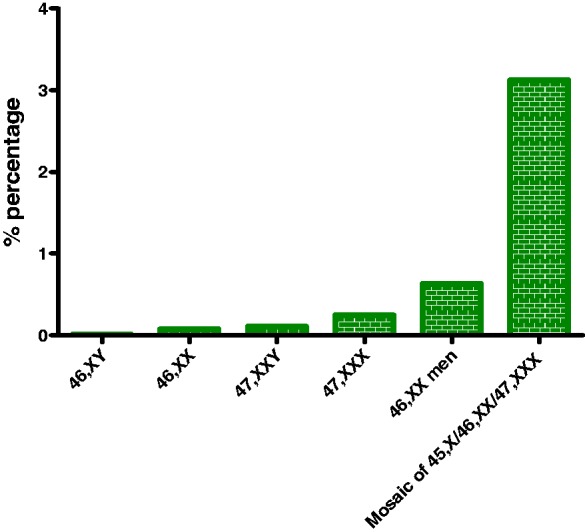
SLE prevalence among individuals with X chromosome abnormalities. Rare X chromosome abnormalities are commoner in SLE than in the general population. X chromosome dosage might have a pathogenetic role in SLE, as the risk for SLE increases along with the number of X chromosomes.

Rare X chromosome abnormalities are much more common in SLE patients than in the general population. Patients with Klinefelter's syndrome (47,XXY) are prone to develop SLE, with the risk being 14-fold higher than in 46,XY men and similar to normal women.^29^ A case of a child with Klinefelter's syndrome that developed SLE and had no detectable sex hormones suggests that the increased expression of X-linked genes might confer susceptibility to SLE.^30^ One in 404 women with SLE reportedly has X trisomy (47,XXX) – which is 2.5 times higher than the reported population prevalence (1/1000) – and exhibits normal sex hormone levels.^31^ Even the extremely rare 46,XX male karyotype has been found to be more frequent in SLE male patients (1 in 316 versus 1 in 20,000–25,000 live male births),^32^ and the same stands true for the triple mosaic 45,X0/46,XX/47,XXX (1 in 800 versus 1 in 25,000 live female births), in which 94% of the cells are 46,XX, and 3% are either 45,X or 47,XXX.^33^ In contrast, Turner's syndrome does not predispose to SLE since only three cases of coexistent SLE and Turner's syndrome (45,X0) have been reported so far.^34^.Together, these data suggest that aberrant X chromosome dosage may have a pathogenetic role in SLE, which is more prevalent in cases of rare X chromosome abnormalities (Figure 1).

Apart from the X chromosome aneuploidies, a deranged number of X chromosome gene copies might have a role in SLE. Chagnon et al. reported a case of an adolescent male who developed SLE at 6 years of age.^35^ He had a Xp22.23;Yp11.2 translocation leading to partial triplication of 12 genes of the Pseudoautosomal Region-1, which are also triplicated in Klinefelter's syndrome patients.^35^ It could therefore be hypothesized that the p arm of the X chromosome may contain genes with a pathogenetic role in SLE.

#### Disturbed X chromosome inactivation process and SLE

In healthy females (46,XX), very early in development, one of the two X chromosome copies becomes at random transcriptionally silenced and inactivated (so-called X chromosome inactivation process, XCI). As a result, women exhibit a mosaic of two cell populations (50% of paternal and 50% of maternal origin). In Klinefelter's syndrome patients (47,XXY) the X chromosome is not randomly inactivated and a preferential inactivation of either the paternal or the maternal X chromosome occurs (X inactivation).^36^ As a result, the patients' cells lose the normal mosaicism, which might impact the production of tolerizing T cells in the thymus, as this skewing could exist in thymic cells, especially in the antigen-presenting cells.^32^ Given that Klinefelter's syndrome is associated with a 14-fold risk of SLE, it remains to be defined whether skewed X inactivation could explain the increased risk of SLE in these patients and generally in patients exhibiting an increased number of X chromosomes.

Under physiologic conditions, XCI is not complete and up to 25% of X-linked genes can be expressed at higher levels in females as compared to males.^37^ This is important as the X chromosome contains many immune-related genes and miRNAs in contrast to the Y chromosome.^3^ As a result, females will have cells with two copies of immune-related X-linked genes, which possibly predispose them to the development of autoimmune diseases such as SLE. Hewagama et al. showed that several immune-related X-linked genes are upregulated in SLE patients and exhibit a female-dominant expression pattern.^38^ For example, CXCR3 (Xq13), OGT (Xq13) and CD40LG (Xq26) are demethylated and overexpressed in CD4 ^+^ T cells of female SLE patients.^38^ FOXP3 (Xp11) has also been found to be significantly increased in CD8^+^ T cells in SLE female patients.^39^ Of importance, it was demonstrated that 18 X-linked miRNAs are overexpressed in T cells of female versus male SLE patients, and oestrogen could potentially influence the expression of 13 miRNAs.^38^ Additionally, a recent research paper proves that TLR7 biallelic expression through evasion of X chromosome inactivation in specific immune cell subsets such as pDCs, B cells and monocytes is observed in women and men with Klinefelter phenotype.^40^ These cells exhibit increased TLR7 expression as expected and TLR7 ligand responsiveness, while especially B cells show higher differentiation tendency to immunoglobulin secreting cells.^40^ It is plausible that reactivation of X-linked genes could explain the high risk of SLE development in healthy females (46,XX) and in individuals with syndromes of increased number of X chromosome copies.

### Microbiota

#### SLE and microbiota

The human microbiota refers to the entire content of microbes that inhabit the human body and is demonstrated to have a key role in human health and disease.^41^ Importantly, a pathophysiological interplay between microbiota and SLE has been recently suggested.

Several microbes have been isolated in biopsies from SLE patients, such as the isolation of *Escherichia coli* in lupus nephritis biopsies, *Enterococcus gallinarum* in liver biopsies, and cell-wall-deficient forms of *Propionibacterium acnes, Corynebacterium* spp., *Staphylococcus epidermidis* and *Streptococcus* spp. in cutaneous lesions of lupus patients.^41,42^ Additionally, several SLE cohorts have demonstrated the existence of gut microbiota dysbiosis. For instance, it has been found that SLE patients exhibit an increase in the Bacteriodetes/Prevotellaceae groups, and a decrease in the Lachnospiraceae/Ruminococcaceae and in Firmicutes/Bacteriodetes ratio.^1^ SLE patients also exhibit dysbiotic subgingival microbiota, with increased proportions of anaerobic bacteria compared to healthy individuals who exhibit aerobic species.^43^ The reported subgingival microbiota dysbiosis can explain, in part, the high rates and the increased severity of periodontitis in SLE patients.^43^ However, it is essential to delineate whether the observed microbiota dysbiosis in SLE patients is a result of the disease process or whether it is the altered microbiota that contribute to the onset and progression of the disease. A study addressing this question supports a direct role of gram-positive pathobionts in the pathogenesis of SLE.^42^ It demonstrates that the use of oral vancomycin or ampicillin in lupus-prone (NZB × BXSB)F_1_ mice decreases mice mortality and the levels of anti-dsDNA and anti-RNA auto-antibodies, and ameliorates the autoimmune manifestations.^42^ *Enterococcus gallinarum* is considered a candidate pathobiont, as it is found to translocate from the gut, to invade systemic organs such as the liver, and to initiate autoimmune manifestations and the production of auto-antibodies in lupus-prone hosts.^42^ Of importance, co-culture of *E. gallinarum* with hepatocytes from healthy livers and (NZB × BXSB)F_1_ mice leads to the production of factors promoting autoimmunity such as β2-glycoprotein 1 (β2GPI) and type I interferon.^42^

The microbial metabolome might also play a role in the pathogenesis of SLE. The microbial dysbiosis leads to gut microbiome alterations, which might have an effect on the immune system via epigenetic dysregulation.^44^ For example, the Lachnospiraceae family strains, which are decreased in SLE, produce butyrate and promote the differentiation of Tregs possibly via epigenetic mechanisms.^41,44^ A better understanding of the microbiome, its role in both gut immunity and the development of systemic inflammation, as well as its metabolic capabilities, is needed.^45^

Some studies support the existence of molecular mimicry to microbial antigens. For example, antiphospholipid antibodies can bind to a homologous β2GPI sequence found in *Haemophilus influenzae* and *Neisseria gonorrhoeae* surface proteins or in the *tetanus* toxoid.^41^ Of note, the anti-dsDNA autoantibodies can bind to the sequence ARVLWRATH of cytochrome B561 and to the sequence RAGTDEGFG of one of the transcription regulators encountered in *Burkholderia* spp.^41^. A recent study took this observation further and demonstrated that several human commensal bacteria encode Ro60 orthologs with high similarity with the corresponding human Ro60, and trigger the production of anti-Ro60 antibodies and Ro60 autoreactive T cells in SLE patients.^46^ The anti-Ro60 antibodies are antinuclear antibodies and are commonly found in SLE, especially in subacute cutaneous lupus erythematosus and in neonatal lupus erythematosus.^46^ This study is very important as it suggests that the microbiota could initiate and sustain an autoimmune disease such as SLE through the cross-reactivity between commensal microbiota and self-antigens, particularly in individuals with a genetic background for autoimmunity such as SNPs in the HLA region.^46^

#### SLE sexual dimorphism and microbiota

Data referring to the contribution of gut microbiota in SLE sexual dimorphism mostly come from mice. Female lupus-prone MRL/lpr mice had significantly higher levels of Lachnospiraceae and Bacteroidetes S24-7, lower *Bifidobacterium* and Erysipelotrichaceae.^47^ A significant decrease in *Lactobacillaceae* and an increase in *Lachnospiraceae* levels, both prior to the onset of the disease and in the late stage of the disease when severe lupus symptoms were noted, suggest that these dysbiosis of these strains might correlate with disease activity in female MRL/lpr mice.^47^ In other studies, gut microenvironment differs between genders and this could play a role in the development of SLE, as the female SNF1 mice have more gut-imprinted α4β7 T cells, higher expression of type I interferons, and a larger number of cells secreting interleukin-17 (IL-17), IL-22 and IL-9.^48^ Additionally, a set of microbiome genes have the ability to metabolize oestrogens into the active forms that enter the bloodstream and act on the oestrogen receptors.^49^ However, the interaction between sex hormones and gut microbiota and to what extent the microbiota-derived sex steroids contribute to the pathogenesis of sexual dimorphism in SLE need to be elucidated.^50^ A summary of the above-mentioned mechanisms is provided in Table 3.

**Table 3:** Pathophysiological mechanisms through which gut microbiota could contribute to female predominance in SLE

Mechanism	Example
Gender-specific differences in microbial dysbiosis and gut microbiota strains	Female MRL/lpr mice: ↑Lachnospiraceae ↑Bacteroidetes S24-7 ↓Bifidobacterium ↓Erysipelotrichaceae ↑ Strain diversity
Gender-specific altered gut microenvironment leading to immunological changes	Female SNF1 mice: ↑α4β7 T cells ↑ Type I IFNs ↑ IL-17-, IL-22-, IL-9-secreting cells
Sex hormones and gut microbiota interaction	Microbiome genes produce active forms of oestrogen

## Concluding remarks

**Figure 2:**
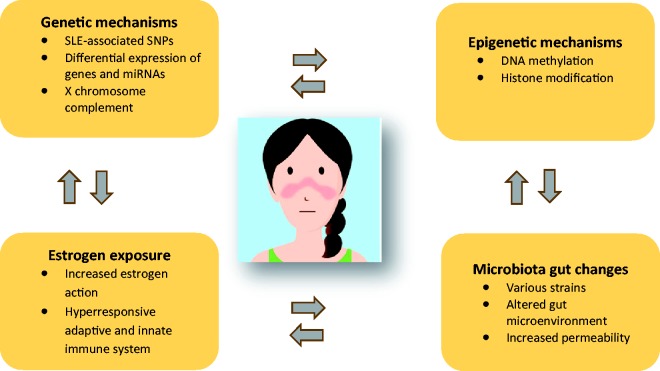
Key candidate pathophysiological contributors to female predominance in SLE.

The striking female predominance and gender-specific disease characteristics represent some of the most interesting aspects of SLE. SLE-associated SNPs in genes important for the immune system and sex hormone signalling along with sex hormone disturbance are considered to play a substantial role (Figure 2). Additionally, the dysregulation of epigenetic mechanisms as well as the X chromosome gene dosage are highly implicated in the development of sexual dimorphism in SLE. Further studies are needed to evaluate whether microbiota dysbiosis plays a role in this sexual dimorphism and to shed light on additional SLE pathogenetic pathways that could be used in advanced personalized therapeutic treatments.
